# Sex-Specific Differences in Autophagic Responses to Experimental Ischemic Stroke

**DOI:** 10.3390/cells10071825

**Published:** 2021-07-20

**Authors:** Anthony N. Patrizz, Jose F. Moruno-Manchon, Lena M. O’Keefe, Sarah J. Doran, Anita R. Patel, Venugopal R. Venna, Andrey S. Tsvetkov, Jun Li, Louise D. McCullough

**Affiliations:** 1 Department of Neurology, McGovern Medical School at the University of Texas Health Science Center at Houston, 6431 Fannin Street, Houston, TX 77030, USA; anthony.patrizz@uth.tmc.edu (A.N.P.); Jose.Felix.MorunoManchon@uth.tmc.edu (J.F.M.-M.); Venugopal.R.Venna@uth.tmc.edu (V.R.V.); andrey.s.tsvetkov@uth.tmc.edu (A.S.T.); Jun.Li.3@uth.tmc.edu (J.L.); 2 Department of Neurology, Beth Israel Deaconess Hospital, 330 Brookline Avenue, Boston, MA 02215, USA; lena.m.okeefe@uth.tmc.edu; 3 Department of Neuroscience, University of Connecticut Health Center, 263 Farmington Avenue, Farmington, CT 06030, USA; dorans2@ucmail.uc.edu (S.J.D.); anitarp@umich.edu (A.R.P.)

**Keywords:** autophagy, middle cerebral artery occlusion, 3-methyladenine, sex differences, ischemic stroke, neuroprotection

## Abstract

Ischemic stroke triggers a series of complex pathophysiological processes including autophagy. Differential activation of autophagy occurs in neurons derived from males versus females after stressors such as nutrient deprivation. Whether autophagy displays sexual dimorphism after ischemic stroke is unknown. We used a cerebral ischemia mouse model (middle cerebral artery occlusion, MCAO) to evaluate the effects of inhibiting autophagy in ischemic brain pathology. We observed that inhibiting autophagy reduced infarct volume in males and ovariectomized females. However, autophagy inhibition enhanced infarct size in females and in ovariectomized females supplemented with estrogen compared to control mice. We also observed that males had increased levels of Beclin1 and LC3 and decreased levels of pULK1 and p62 at 24 h, while females had decreased levels of Beclin1 and increased levels of ATG7. Furthermore, the levels of autophagy markers were increased under basal conditions and after oxygen and glucose deprivation in male neurons compared with female neurons in vitro. E2 supplementation significantly inhibited autophagy only in male neurons, and was beneficial for cell survival only in female neurons. This study shows that autophagy in the ischemic brain differs between the sexes, and that autophagy regulators have different effects in a sex-dependent manner in neurons.

## 1. Introduction

Epidemiologic and clinical evidence have demonstrated the importance of sex differences in the incidence and response to ischemic brain injury [1,2,3]. Women have lower stroke incidence relative to men until well after menopause; however, rates climb dramatically in elderly women who also have greater disability, morbidity and mortality after stroke than men [2,4]. Previous studies have suggested that sex-dependent pathways are activated in response to stroke, including caspase-dependent apoptosis and poly (ADP-ribose) polymerase-mediated DNA repair [5,6,7,8]. With the cost of stroke care in the USA projected to exceed 180 billion dollars by 2030, understanding sex differences and optimizing neuroprotective agents is critical to the development of efficacious therapies [9,10,11]. 

Macroautophagy (called autophagy thereafter) is a self-catabolic process where subcellular proteins, macromolecules, and organelles are sequestered within membrane-enclosed vesicles (autophagosomes) and are degraded by fusion with lysosomes (autolysosomes) [12,13,14,15]. Autophagy plays a role in cellular homeostasis by degrading damaged cellular contents and redistributing the constituents for other cellular processes [14]. During times of cell stress, such as ischemia, autophagy may become dysregulated and increase injury, or conversely may increase the ability of the cell to survive under conditions with low energy substrates. There is increasing evidence that autophagy is a sex-dependent process [13,16,17,18].

Recent reports have revealed increased levels of autophagy in experimental models of both hemorrhagic and ischemic stroke [19,20,21,22,23]. Pharmacological inhibition of autophagy in male animals during or up to several hours after experimental stroke reduced tissue death [19,20,24,25,26,27]. Studies in neonates show that females have higher basal levels of autophagy and caspase activation following hypoxic-ischemic injury [28]. We hypothesized that sex differences are present in autophagy in the ischemic brain, and that down regulating autophagy after experimental ischemic stroke in mice would have differential efficacy in males and females.

## 2. Materials and Methods

### 2.1. Animals

C57BL/6J mice were purchased from Harlan Laboratories. All experiments were performed according to NIH guidelines for the care and use of animals in research and under protocol #100442-0515 approved on 30 August 2012 by the University of Connecticut Health Center Institutional Animal Care and Use Committee. 118 young mice (8–12 weeks, 21–27 g) of both sexes were used. 20 mouse pups of both sexes were used to generate the neuronal cultures.

### 2.2. Transient Cerebral Ischemia Model

Focal transient cerebral ischemia was induced by middle cerebral artery occlusion (MCAO) for 90 min followed by reperfusion as described previously [29,30]. Sham mice underwent the same procedure but the silicon filament was not advanced through the internal carotid. All animals were fed with wet mash and administered subcutaneous saline injections following surgery until sacrifice [31]. Necropsy was performed on all mice after sacrifice to exclude any animals with evidence of subarachnoid hemorrhage. All animals were randomly assigned to treatment group.

### 2.3. 3-Methyladenine (3MA) Administration

3MA (162 nmol in 2 µL dimethyl sulfoxide (DMSO); M9281, Sigma–Aldrich, St. Louis, MO, USA) or vehicle (2 µL DMSO, D8418, Sigma-Aldrich) was administered by intracerebroventricular injection 30 min following MCAO as described previously with modifications. [32] A burr hole was made with the tip of a 30 gauge needle in the right skull at the coordinates −0.9mm lateral and −0.1mm posterior from Bregma, followed by needle insertion of a 10 µL, 33 gauge syringe (Model 701 SN, Hamilton Company) to a depth of −3.1mm. 2 µL of 3MA in DMSO or DMSO vehicle was injected over 2 min. The dose was selected based on previous reports in rat models of MCAO and subarachnoid hemorrhage using 600 nmol [19,23]. The dose was adjusted using the ratio of brain size between Sprague-Dawley rats and C57BL/6 mice, calculated at 0.27 [33,34]. 3MA was dissolved in DMSO by heating the solution to 99 °C for 2–4 s and allowing it to cool to room temperature prior to injection.

### 2.4. Ovariectomy and Estrogen Supplementation

Ovariectomy was performed 10 days prior to MCAO. Estradiol was administered in subcutaneous capsule containing 17β-estradiol (180 µg/mL; Sigma–Aldrich, St. Louis, MO, USA) in sesame oil beginning 10 days before MCAO [35]. Uterine weights were obtained at sacrifice [8].

### 2.5. Histological Assessment

Mice were anesthetized with a 0.1 mL/10 g body weight dose of Avertin (T48402, Sigma-Aldrich, St. Louis, MO, USA) dissolved in 2-Methyl-2-Butanol 72 h after MCAO. Animals were perfused transcardially with phosphate-buffered saline followed by 4% paraformaldehyde. The brain was removed from the skull, post-fixed for 24 h, and subsequently placed in cyroprotectant (30% sucrose). The brain tissue was cut into 30-μm free-floating coronal sections and was stained using cresyl violet (CV) (C5042, Sigma-Aldrich, St. Louis, MO, USA) for evaluation of ischemic cell damage [36]. Images were acquired by a charge-coupled device camera (Micropublisher 5.0 RTV, QImaging, Tucson, AZ, USA) and analyzed using Sigmascan Pro5 (Systat Software Inc., San Jose, CA, USA) as described [36]. We calculated the infarct volume by imaging eight slices spanning the territory of the MCA. The contralateral hemisphere was used to correct for volume and edema. Infarct volumes were expressed as a percentage of the ipsilateral hemisphere. Animals were excluded if they had a posterior cerebral artery occlusion, no intra-ischemic deficits, or had a surgical complication such as subarachnoid hemorrhage. Exclusions included: vehicle treated females: 3; 20%; drug treated females: 3; 20%; vehicle treated males: 4; 36%; drug treated males: 2; 16%; vehicle treated oil supplemented ovariectomized females (OVX): 3; 17%; drug treated oil supplemented OVX females: 2; 15%; vehicle treated E2 supplemented OVX females (OVX + E2): 2; 18%; drug treated OVX + E2: 2; 22%. Investigators were blinded to treatment group, and random animals were examined by multiple investigators.

### 2.6. Subcellular Fractionation

After 6 and 24 h post MCAO, brain samples were obtained by rapid removal of the brain, resection of the cerebellum and olfactory bulbs, and immediate dissection into the right (R; ischemic) and left (L; non-ischemic) hemispheres. Samples were homogenized in 1.5 mL of ice-cold RIPA (Cell Signaling, Danvers, MA, USA) buffer containing protease inhibitor tablet (Roche, Indianapolis, IN, USA), PhosSTOP (Roche, Indianapolis, IN, USA), and 1 mM PMSF using a Dounce homogenizer on ice and followed by brief sonication on ice. Homogenates were separated into cytosolic, mitochondrial, and nuclear fractions as described [37].

### 2.7. Western Blot

The protein concentration of the fractionated samples was determined by BCA Protein Assay Kit (Thermo Fisher Scientific, Waltham, MA, USA). 15 µg of cytosolic proteins were loaded per lane in a 7.5% SDS/PAGE gel (TGX Criterion, transferred to a PVDF membrane, blotted with 5% Bovine Serum Albumin (BSA), and probed with antibodies for ATG7 (anti-rabbit, 1:1000 Thermo Fisher Scientific, Waltham, MA, USA), Beclin1 (anti-rabbit, 1:500, Cell Signaling Technology, Danvers, MA, USA), phospho-ULK1 (pULK1) (anti-rabbit, 1:1000, Cell Signaling Technology, Danvers, MA, USA), LC3-II (anti-rabbit 1:500, Cell Signaling Technology, Danvers, MA, USA), p62 (anti-mouse, 1:1000 Cell Signaling, Danvers, MA, USA), and antibody for Beta-Actin (1:10,000, Sigma Aldrich, St. Louis, MO, USA) was used as a loading control. Blots were incubated with ECL signal detecting reagent (Thermo Scientific, Waltham, MA, USA) and developed using Bio Rad ChemiDoc XRS+ and densitometry was performed using Image Lab software version 5.

### 2.8. Neuronal Culture

Cortices from individual embryonic (E20) rats were dissected, digested with papain (5 µL/mL, LS003127, Worthington, Lakewood, NJ, USA), treated with trypsin inhibitor (1.5 mg/mL, T9253, Sigma, St. Louis, MO, USA) solutions, and homogenized. 500,000 neurons per well were plated in 24-well plates previously coated with poly-D-lysine (1 mg/mL, A-003, Millipore, Burlington, MA, USA) and maintained with NeuroBasal Media supplemented with B27 (17504001, Thermo Fisher Scientific, Waltham, MA, USA), GlutaMax Supplement (35050061, Thermo Fisher Scientific, Waltham, MA, USA), and penicillin-streptomycin (100 U/mL, 15140122, Thermo Fisher Scientific, Waltham, MA, USA) [38,39,40]. The sex of the embryos was determined by agarose gel electrophoresis using PCR primers for the Y chromosome marker gene, Sry [41]: Sry-FW, 5-AAGCCTTACAGAAGCCGAAAAA-3; Sry-RV, 5-TGTGGCACTTTAACCCTTCGA-3 (Supplemental Figure S1).

For western blotting, neurons were maintained for 14 days. Neurons were pre-treated with vehicle (100% ethanol) or E2 (20 nM, E8875, Sigma, St. Louis, MO, USA) for 1 h. Cells were washed with PBS and incubated with complete media (normoxia condition) or subjected to oxygen and glucose deprivation (OGD/R; 140 mM NaCl, 35 mM KCl, 17 mM CaCl_2_, 12 mM MgSO_4_, 0.4 mM KH_2_PO_4_, 5 mM NaHCO_3_, and 10 mM HEPES, pH 7.4) in a hypoxia incubator chamber for 1 h at 37 °C. Cells were then washed with supplemented Neurobasal media and incubated with the same media for 2 h.

### 2.9. Longitudinal Imaging and Survival Analysis

Primary neurons cultured from individual embryos and at 4 days in vitro, (DIV) were transfected with the DNA construct pCAG-GFP to visualize neuronal morphology using Lipofectamine 2000 Transfection Reagent (11668060, Thermo Fisher Scientific, Waltham, MA, USA), as previously described [42,43,44]. pCAG-GFP was a gift from Connie Cepko (Addgene, 11150). After transfection, neurons were maintained until 14 days in vitro to mature and then treated with vehicle or E2 and incubated in normoxia or OGD/R conditions as described above. Cells were immediately imaged after treatments with the longitudinal imaging system EVOS FL Auto 2, which allows us to track the same group of neurons over time [42,43,44]. We imaged cells every 24 h for 5 days. Neurons that died during the imaging interval were assigned a survival time, which refers to the period between transfection and their disappearance from an image. These event times were used to obtain the exponential cumulative survival graphs and analyzed for statistical significance by Log-Rank test. We used the JMP (SAS Institute, Cary, NC, USA) software for statistical analyses and curves generation. Note that transfection efficiency in cultured primary neurons was less than 5%. Thus, less than 25,000 neurons per 24-well plate were transfected. However, only 100 neurons per well and treatment were required for survival analyses purposes. 

### 2.10. Statistics

Statistics are presented as mean ± standard deviation for all experiments. Statistics were performed using GraphPad Prism. Interval power analysis was performed to determine group size. A two way ANOVA was performed when comparing multiple groups. A probability value of *p* < 0.05 was considered statistically significant. If an interaction was statistically significant, then Sidak’s post-hoc analysis was used to assess where the interaction occurred. If there was no interaction statistical significance, main effects were reported if there was statistical significance. Neuronal survival between groups was analyzed with the Log-Rang test using the JMP software. All investigators were blinded to surgical condition when analyzing data.

## 3. Results

### 3.1. Sex Differences in Infarct Size Following Administration of 3MA

**Figure 1:**
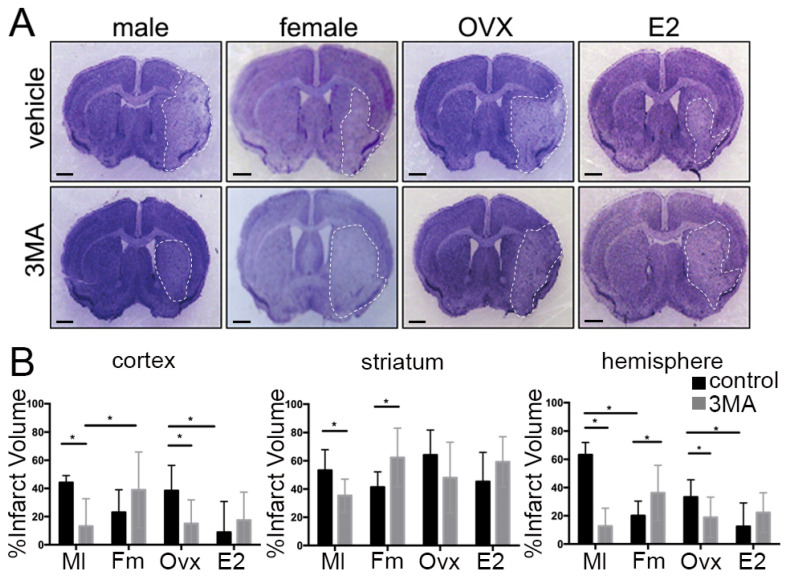
Infarct size following 3MA treatment. (**A**) Representative cresyl violet staining of 3MA and vehicle-treated male, female, OVX and E2 supplemented stroke mice 72 h after stroke onset. Scale bar, 1 mm. (**B**) Quantification of infarct volume in cortex, striatum, and hemisphere. * Represents statistical significance (*p* < 0.05).

To determine if differences exist in the pathophysiology of ischemic injury by inhibiting autophagy between male and female mice, we measured the infarct volume 72 h after MCAO and after administration of either 3MA in DMSO or DMSO vehicle-control. We confirmed that the levels of the autophagy marker LC3 were reduced in 3MA-treated mice 72 h after MCAO (vehicle vs. 3MA, *p* = 0.0203), supporting the inhibitory effect of 3MA on autophagy in the brain (Figure S2). Representative CV stained sections are shown in Figure 1A. There was a statistical difference in the effect of 3MA treatment on cortical (F(1,33) = 12.82, *p* < 0.01), striatal (F(1,33) = 18.68, *p* < 0.01) and hemispheric infarct volume (F(1,33) = 51.71, *p* < 0.0001, *n* = 7/male vehicle, 10/male 3MA, 8/female vehicle, 12/female 3MA) (Figure 1B). 3MA-treated males had reduced infarct volume when compared to male vehicle-controls (cortex *p* < 0.05; striatum *p* < 0.05 and hemisphere *p* < 0.001; Sidak’s test for multiple comparisons). In contrast, there was no benefit of 3MA treatment in females. Administration of 3MA led to a significant increase in infarct volume in the striatum (*p* = 0.001); as well as in the hemisphere (*p* < 0.05). Previously, we demonstrated that cerebral blood flow does not differ between female and male mice in this model of ischemic stroke [45,46].

### 3.2. The Effect of Ovarian Hormones on Autophagy

To control for the known neuroprotective effect of estrogen on ischemic stroke, female mice were ovariectomized and supplemented with either sesame oil or 17β-estradiol ten days prior to MCAO surgery as previously described [35]. Representative CV stained sections are shown in Figure 1A. OVX vehicle-control mice had infarct volumes similar to vehicle-control males and had larger infarcts than ovary-intact vehicle-control females, consistent with the known neuroprotective effects of estrogen. There was a statistically significant effect of hormone supplementation and 3MA treatment on infarct volumes in cortex (F(1,37)= 6.927. *p* < 0.05, *n* = 14/OVX vehicle, 11/OVX 3MA, 9/E2 vehicle, 7/E2 3MA), striatum (F(1,37) = 5.095, *p* < 0.05), and hemisphere (F(1,37) = 7.09, *p* < 0.05) (Figure 1B). OVX mice that received 3MA had a reduction in infarct volume compared to vehicle-controls in the cortex (*p* < 0.01), and in hemisphere (*p* < 0.05), similar to the treatment outcome seen in 3MA-treated males. 3MA-treated OVX + E2 had an increase in infarct volume in all brain areas when compared to sham; however, this increase was not statistically significant.

### 3.3. Sex Differences in Autophagy Protein Levels at 6 h

**Figure 2:**
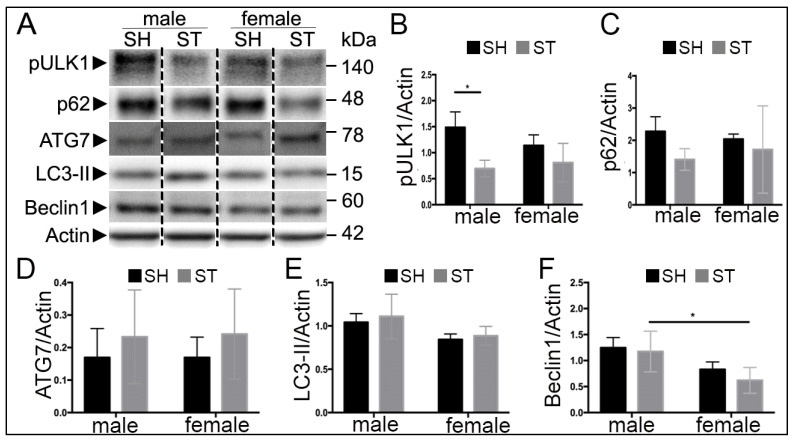
Autophagy proteins 6 h after ischemia. (**A**) Representative Western blots of Beclin1, pULK, LC3, and ATG7. (**B**) Quantification of pULK1. An effect of stroke was observed, (*p* = 0.001) with lower levels of pULK1 observed in stroke males compared to sham. (**C**,**D**) No observable differences in levels of p62 and ATG7. (**E**) Quantification of LC3. An effect of sex was observed with males displaying increased levels, (*p* = 0.01). (**F**) Quantification of Beclin1. An effect of sex was observed (*p* = 0.003) with male strokes displaying higher levels compared to female strokes. * *p* < 0.05.

To examine the effect of sex on autophagy activation following cerebral ischemia, MCAO was performed, and brain levels of Beclin1, pULK1 (phosphorylated at SER757), LC3, p62 and ATG7 were measured 6 h following reperfusion in young males and gonadal-intact females. Representative western blots are shown in Figure 2A and quantified: pULK1 (Figure 2B), p62 (Figure 2C), ATG7 (Figure 2D), LC3-II (Figure 2E) and Beclin1 (Figure 2F).

Stroke decreased the levels of pULK1 in male (F(1,12) = 17.06, *p* < 0.01, *n* = 4 stroke, *n* = 3 sham) (Figure 2B). Males displayed increased levels of LC3 (F(1,12) = 7.8, *p* = 0.01) (Figure 2E) and levels of Beclin1 compared to females (F(1,11) = 14.3, *p* < 0.05) (Figure 2F). There was no significant effect of sex or stroke on levels of p62 or ATG7.

### 3.4. Sex Differences in Autophagy Protein Levels at 24 h

**Figure 3:**
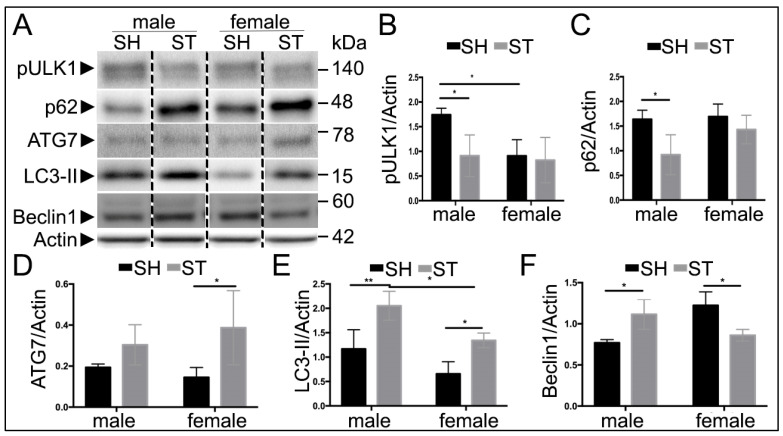
Autophagy proteins 24 h after ischemia. (**A**) Representative Western blots of Beclin1, pULK1, LC3, and ATG7. (**B**) Quantification of pULK1, effect of both sex and stroke (*p* < 0.05 for each), with male strokes displaying lower levels of pULK1 compared to sham. (**C**) There is an effect of stroke on the levels of p62 (*p* = 0.01) while there is a decrease in p62 following stroke in males (*p* < 0.05). (**D**) Effect of stroke on levels of ATG7 (*p* = 0.01) showing increased levels in female stroke compared to sham. (**E**) Quantification of LC3, there was both an effect of sex (*p* < 0.01) and stroke (*p* < 0.001), but no interaction (*p* = 0.52). Levels of LC3 increased following stroke in both males (*p* < 0.01) and females (*p* < 0.05) compared to their sham counterparts, with stroke males displaying higher levels than stroke females (*p* < 0.05). (**F**) Quantification of Beclin1 revealed an interaction of sex and stroke (*p* = 0.001) with an increase in male stroke compared to male sham as well as a decrease in female stroke compared to female sham. * *p* < 0.05, ** *p* < 0.01.

The pathophysiology of stroke evolves with time, and thus the autophagic response may change as well. To assess this, protein levels of Beclin1, pULK1, LC3, p62 and ATG7 were also measured at 24 h following reperfusion by western blot (Figure 3A), at a time when the histological damage is complete in this model. (Liu, et al., 2009b) There was no interaction of stroke and sex on levels of pULK1, however, there were effects of stroke (F(1,10) = 5.131, *p* < 0.05) and sex (F(1,10) = 5.165, *p* < 0.05, *n* = 4 stroke and = 3 sham) (Figure 3B). In sham animals, males displayed higher levels of pULK1 than females (*p* < 0.05). Stroke males had lower levels of pULK1 than sham (*p* < 0.05), whereas stroke females displayed similar levels to sham. There was an effect of stroke on the levels of p62 (F(1,10) = 8.831, *p* = 0.01) (Figure 3C), additionally, the levels of p62 decreased following stroke in males (*p* < 0.05). Unlike the 6-h findings, levels of ATG7 showed an effect of stroke (F(1,10) = 8.06, *p* = 0.01), with stroke females exhibiting higher levels compared to sham (*p* < 0.05) (Figure 3D). There was both an effect of sex (F(1,10) = 16.65, *p* < 0.01) and stroke (F(1,10) = 27.64), *p* < 0.001) on levels of LC3-II, but no interaction (*p* = 0.52). Levels of LC3 were increased following stroke in both males (*p* < 0.01) and females (*p* < 0.05) compared to their sham counterparts, with stroke males displaying higher levels than stroke females (*p* < 0.05) (Figure 3E). There was an interaction of stroke and sex on levels of Beclin1 (F(1,8) = 22.7, *p* = 0.001) (Figure 3F). Stroke males had increased levels of Beclin1 compared to sham (*p* = 0.02), while stroke females exhibited decreased levels of Beclin1 compared to sham (*p* = 0.01). IHC was performed to determine the spatial distribution of LC3 and p62 in the stroke brain, revealing co-localization of p62 and LC3 in the peri-infarct cortical regions of the ipsilateral hemisphere (Figure S3).

### 3.5. Sex Differences in Autophagy Markers Following E2 Treatment and Oxygen and Glucose Deprivation and Reperfusion (OGD/R)

**Figure 4:**
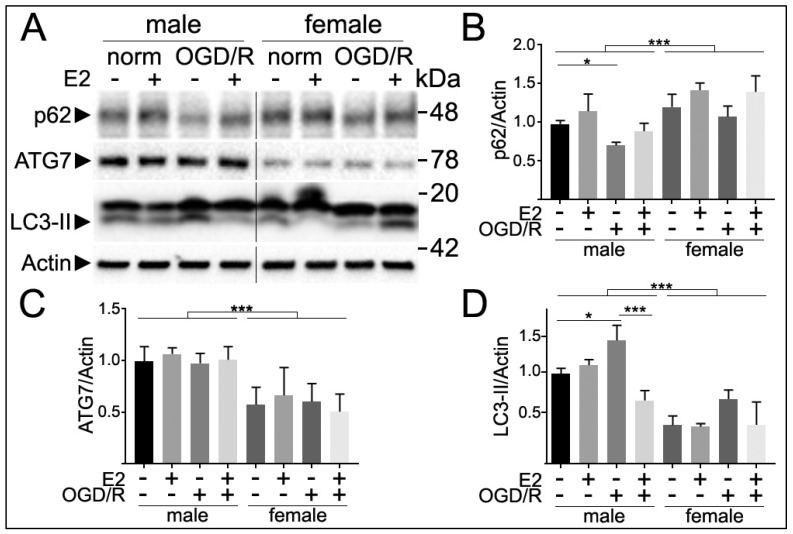
Sex differences in autophagy markers. (**A**) Cultured primary neurons from male and female embryonic rat pups (14 DIV) were pre-treated with 17β-estradiol (E2) or a vehicle (ethanol) for 1 h and then subjected to oxygen and glucose deprivation (OGD) or normoxia (norm) for 1 h. Complete media was restored (reperfusion, /R) for 6 h after treatments and cells were collected and processed for analyses of p62 and Atg7 levels by immunoblotting. (**B**) Quantification of p62 showed enhanced levels by sex (*p* < 0.0001) without any interaction (*p* = 0.399). Neurons from females exhibited enhanced levels of p62 compared with male neurons. OGD/R reduced levels of p62 compared with normoxia (norm) conditions in males but not in females. (**C**) Quantification of ATG7 showed reduced levels in female neurons compared with male neurons (*p* = 0.0003) independently of treatments (*p* = 0.606). No interaction was observed (*p* = 0.962). (**D**) Quantification of the LC3-II band intensities showed enhanced levels by sex (*p* < 0.0001) and treatments (*p* < 0.0001) with interaction (*p* = 0.014). Neurons from females exhibited reduced LC3-II levels. Post-hoc Tukey analyses were performed after two-way ANOVA analyses. * *p* < 0.05, *** *p* < 0.0001. Results were pooled from 4-5 male and 4-5 female embryonic (E20) pups.

The cerebral cortex consists of approximately 30% neurons [47] and the remaining glial cells can also regulate neuronal autophagy [48]. To explore sex differences in autophagy in neurons selectively, we examined levels of p62 and ATG7 in primary neuronal cultures. p62 is considered an autophagy flux marker [49,50,51,52] and ATG7 participates at the early stages of autophagy during membrane extension [53,54,55]. We pre-treated cultured neurons with vehicle or E2 and then subjected cultures to normoxia or OGD/R conditions. We analyzed p62, ATG7 and LC3-II levels by western blotting. p62 levels were increased in female neurons compared with males (F(1,24) = 18.4, *p* < 0.0001, *n* = 5/grp), suggesting that autophagy flux was reduced in cultured neurons isolated from female rat pups. No interaction of sex and treatments was observed (F(3,24) = 2.1, *p* = 0.13). Post-hoc Tukey analyses revealed that p62 levels were significantly reduced after OGD/R in male neurons (*p* = 0.0309), but not in female neurons (*p* = 0.999) (Figure 4). This suggests that autophagy in cultured female neurons is less responsive to OGD/R compared with male neurons.

On the other hand, ATG7 levels were significantly reduced in cultured neurons from embryonic female rats compared with male neurons (F(1,24) = 20.9, *p* = 0.0003), independently of treatment (F(3,24) = 0.6, *p* = 0.606), and no interaction between sex and treatments was observed (F(3,24) = 0.1, *p* = 0.962). There were no significant differences in ATG7 levels with the different treatments (E2 and OGD) in either male or female neurons, indicating that ATG7 levels are not regulated by these external stimuli in cultured neurons.

In addition, LC3-II levels were significantly reduced in cultured primary neurons from female pups compared with male neurons (F(1,24) = 142.47, *p* < 0.0001). Post-hoc Tukey analyses indicated that LC3-II significantly increased with OGD compared with normoxia conditions (*p* = 0.0092), and the E2 treatment significantly reduced OGD-induced LC3-II levels (*p* < 0.0001) in male, but not in females (*n* = 4/grp).

Overall, our data suggest that the autophagy flux is reduced in female neurons compared with male neurons following OGD.

### 3.6. Sex Differences in Neuronal Survival Following E2 Treatment and OGD/R

**Figure 5:**
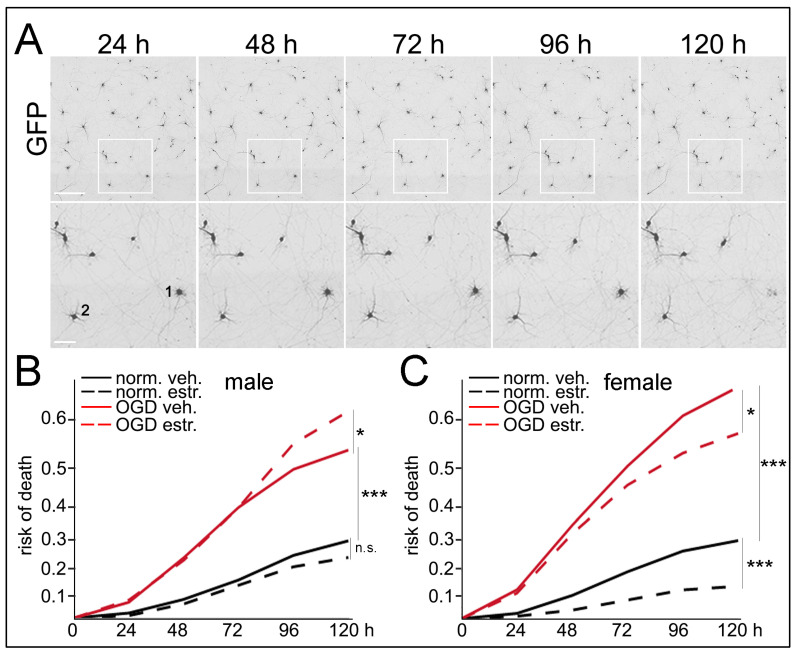
Sex differences in neuronal survival after E2 treatment and OGD/R. (**A**) The top panel is a representative figure of longitudinal imaging of the same group of neurons transfected with GFP (black and white images were color inverted to increase contrast). Scale bar, 400 µm. The bottom panel shows zoomed in GFP-positive neurons that degenerate over time (i.e., neuron 1) or remain alive at the end of the experiment (i.e., neuron 2). Images were inverted and converted to black and white images to increase contrast of GFP-positive neurons. Scale bar, 50 µm. (**B**,**C**) Cultured primary neurons from male (**B**) and female (**C**) embryonic rat pups were transfected with GFP at 4 DIV and let develop for 10 days. Survival graphs of male neurons (**B**) and female neurons (**C**), pre-treated with vehicle (veh.) or with 17β-estradiol (E2) and subjected to normoxia (norm.) or oxygen and glucose deprivation (OGD/R) conditions. OGD/R significantly enhanced neuronal death in both sexes (*p* = 0.0001). Neuroprotective effects of E2 under basal conditions was more evident for female neurons (*p* = 0.0001) than male neurons (*p* = 0.1508). E2 was also protective for female neurons (*p* = 0.0268) after OGD/R, but not for male neurons. * *p* < 0.5, *** *p* < 0.0001, n.s., non-significant (log-rank test). One hundred neurons were analyzed per condition. Results were pooled from 5 male and 5 female embryonic (E20) pups.

The stimuli that regulate autophagy might also determine the neuronal fate [56,57,58]. To study sex differences in cell survival of neurons treated with E2 and OGD, we used a longitudinal imaging system [42,43,44]. Primary neurons were cultured from individual rat embryos, transfected with GFP and maintained for two weeks. Male and female neurons were then treated with vehicle or E2 and subjected to normoxia or OGD/R. GFP-positive neurons were longitudinally imaged for 5 days. We observed that OGD/R induced neuronal death in neurons from embryonic female and male rats (*p* (♀norm vs. OGD) = 0.0001; *p* (♂norm vs. OGD) = 0.0001, *n* = 5/grp) (Figure 5 and Figure S4). Interestingly, while E2 did not affect neuronal survival in male neurons under basal conditions (p (♂norm vs. norm-E2) = 0.1508) and did not mitigate neuronal death induced by OGD/R in male neurons (*p* (♂OGD vs. OGD-E2) = 0.0475, however, E2 had beneficial effects on neurons from females under basal conditions (*p* (♀norm vs. norm-E2) = 0.0001) and significantly reduced OGD/R-induced neurotoxicity in female neurons (*p* (♀OGD vs. OGD-E2) = 0.0268) (Figure 5 and Figure S4). Taken together, we conclude that OGD enhanced neuronal death in both sexes and that under basal conditions and after OGD/R, E2 is differentially protective for female neurons compared to male-derived neurons.

## 4. Discussion

It is well established that neuronal tissue is rapidly lost as stroke progresses. Autophagy plays a major role in the response to cellular stress, and has been implicated in the response to tissue ischemia [59]. Evidence suggests that survival of the ischemic penumbra depends strongly on the interaction between regulators of autophagy and apoptosis [60]. This study provides the first evidence that sex differences in autophagy previously noted in vitro are present in vivo following stroke in adult mice. Five key autophagy proteins, p62, LC3, ATG7, Beclin1, and pULK1, show differential expression in response to cerebral ischemia in males and females. Furthermore, post-stroke treatment with 3MA reduced tissue death in males but had no benefit in females, and exacerbated injury. This has important implications for the development of autophagy modulators as neuroprotective agents.

Down regulation of autophagy by 3MA occurs through interaction with a complex consisting of Vps34, p150, and Beclin1 [61]. A recent systematic review analyzed the effects of 3MA on animal models of cerebral infarction [62]. Unfortunately, there is no consensus about the beneficial effects of 3MA on cell viability in the brain: some studies found that inhibiting autophagy with 3MA prevents cell death after ischemic stroke [27,63]; however, other studies propose that 3MA may promote neuronal death [64,65]. However, none of these examined sex-specific effects or were performed only with male animals. Our study provides evidence about sex differences in brain damage and autophagy after ischemic stroke in mice. We found that at 6 h, there were higher levels of Beclin1 in males when compared to females; however, there was no effect of stroke. At 24 h, stroke males had a significant increase in Beclin1 compared to male shams, whereas stroke females had a significant decrease in Beclin1 compared to female shams. The increase in Beclin1 and LC3-II seen in males suggests that males rapidly induce autophagy after ischemia and could explain the selective effectiveness of 3MA treatment in males after stroke. Coinciding with an increase in Beclin1 and LC3-II levels, male mice subjected to stroke had a decrease in levels of p62, suggesting that the autophagy cargo p62 is degraded and autophagy is stimulated. Further, this suggestion is supported by reduced phosphorylation of ULK1 at SER 757, which is phosphorylated by mTOR and is an indicator of enhanced autophagy [66,67]. The number of studies on sex differences in autophagy in animal models of ischemic stroke is limited. To our knowledge, only one study examined sex differences in autophagy, which examined the autophagy regulator, HIF-1α, which was upregulated in male rats 24 h after ischemic stroke compared with females [68]. Thus, our study provides novel data and highlights the importance of studying the mechanisms that govern sex differences in autophagy in the brain after stroke.

There were no differences in pULK1 in females at either time point. At 24 h, levels of Beclin1 were decreased in stroke females compared to sham, and levels of ATG7 were elevated in the stroke females compared to sham. In vitro studies in neurons have shown that Beclin1-independent autophagy is mediated by ATG7 and leads to caspase-dependent apoptosis [69]. Females may utilize alternative pathways for autophagy induction that do not involve Beclin1 or pULK1 [61,70], or may not utilize induction of autophagy at all [71]. Previous work has shown that while both males and females increase levels of LC3 as a marker for rate of autophagy induction following transient MCAO [68], female neurons undergoing starvation mobilize fatty acids and rely less on autophagy for survival than neurons derived from males [72].

To distinguish autophagic cell death from necrotic or apoptotic cell death, there needs to be an absence of activation of machinery and pathways involved in apoptosis, such as caspase activation [73]. We have previously shown that males do not respond to treatment with a pan-caspase inhibitor, but female mice had significant reductions in infarct with treatment [74]. This further supports the possibility that females use a Beclin1-independent pathway mediated by ATG7 to induce caspase-mediated cell death. Furthermore, poly(ADP-ribose) polymerase-1 (PARP-1) inhibition reduced infarcts in males but exacerbated injury in females in a transient stroke model, again demonstrating the importance of examining both sexes [8]. PARP-1 activation promotes autophagy [75], and “male-specific ischemia” may induce both PARP-1 and autophagy. Either inhibition of PARP or autophagy leads to reduction in ischemic injury selectively in males.

The brain is composed of many non-neuronal cells [47], which can determine neuronal autophagy and neuronal homeostasis [48]. Our data from homogenates of whole mouse brains can underestimate the contribution to autophagy of each cell type, with different cell types regulating autophagy differently [48,76]. For example, sphingosine kinase-1-dependent autophagy is differently regulated between neurons, astrocytes, and cell lines [44,77]. Thus, as an approach to study neuronal autophagy without the influence of other cells types, we analyzed autophagy markers in cultured primary neurons from the cortex of rat embryos and observed that autophagy appears to be reduced in female neurons compared with males. We measured the levels of p62, which serves as a link between the inner membrane of autophagosomes and the sequestered material [49,50]. When autophagosomes fuse with lysosomes p62 is degraded. Thus, higher levels of p62 suggest that cells could not digest p62 by autophagy [51,52]. We found that p62 levels were elevated in female neurons compared with male neurons at basal conditions. OGD/R reduced p62 levels in males, suggesting that OGD/R stimulated autophagy flux in a sex-dependent manner. We also found that ATG7 levels were reduced in female neurons independently of treatments. In addition, the LC3-II levels were significantly reduced in female neurons compared with male neurons, supporting the general assumption that autophagy is reduced in females versus males.

Nutrient deprivation and gonadal hormones are important regulators of autophagy. We wondered if those stimuli determine the neuronal fate in a sex-dependent manner. On one hand, we observed that OGD/R induced cell death in neurons from both sexes. We did not find significant differences between sexes in OGD/R conditions. Sharma et al. [78] found that neurons from female mice were more vulnerable to OGD conditions than male neurons. However, other studies concluded that female neurons were more resistant to OGD [79,80]. This discrepancy can be due to the different methodology used in these studies. Sharma et al. performed 1.5 h OGD instead of 2 h [79]. We found that OGD longer than 1 h dramatically reduced neuronal viability in our system (more than 90% of cells died 24 h after OGD/R). 1 h OGD was more suitable for our longitudinal analyses purposes, since that conditions allowed us to track neuronal death for longer periods in living neurons instead doing analyses only at 24 h after OGD. On the other hand, we observed that E2 was more beneficial for female neurons compared with males. Johnsen and Murphy observed that E2 attenuated isofluorane preconditioning-induced protection from OGD in female but not in male neurons [80]. The design of the mentioned study is different from ours, but this evidence supports our finding that sex differences exist in neuronal survival in response to E2. However, more studies are needed to determine how sex influences autophagy-dependent cell survival in different brain cells. Importantly, our data from longitudinal analyses in cultured primary cortical neurons (Figure 5) mirror our in vivo data. The infarct volume in the cortex of ovariectomized females after stroke was not different from the infarct volume observed in control males (Figure 1B). In addition, E2 treatment reduced the infarct volume in ovariectomized females compared with males, as we observed that E2 was protective for female neurons.

The translational relevance of these findings becomes clear in our pharmacological studies when males and females were treated with 3MA. Unlike males, which were protected by 3MA, females treated with 3MA displayed significantly larger infarcts than vehicle-treated controls. Interestingly, 3MA-treated OVX females demonstrated a reduction in infarct compared to vehicle-controls, suggesting a more “male-like” pattern in the setting of low estrogen. 3MA-treated OVX + E2 mice had an exacerbation of ischemic injury, mimicking what was seen in gonadal-intact females. Thus, these novel in vivo studies are consistent with previous in vitro studies.

There are several limitations to consider when interpreting the results of this study. Importantly, this study only evaluated the role of autophagy in the acute response to cerebral ischemia. Autophagy protein levels and tissue response to 3MA were assessed at or before 72 h, which is days to weeks before the replacement of the infarcted region of brain with scar tissue [81]. The time course of autophagy during injury resolution remains to be explored, and evaluation of proteins involved in autophagy at time points beyond 6 and 24 h could provide insight into the role of autophagy in recovery. The induction of autophagy may also differ based on the stroke model used. The reperfusion model used here is quite severe, but mimics one of the most common clinical strokes. Additionally, ovariectomy induces an abrupt loss of gonadal hormones, which does not perfectly recapitulate the post-menopausal state that occurs with senescence. Numerous studies have reported a decrease in autophagy processes that accompany aging [82]. Interestingly, aging has been shown to alter hormonal regulation of autophagy, i.e., glucagon’s stimulatory effects on autophagy are blunted [83]. Our study is also limited by a lack of evaluation of lysosome function. The lysosome-associated protein LAMP1 accumulates and lysosomal storage is altered in the brains of ischemic mice and in cultured neurons after OGD [84]. Importantly, several genes encoding for proteins associated to lysosomes are linked to the X chromosome (i.e., LAMP2, RAB genes, ATP6AP2, and GLA) [85]. Given that inactivated X-linked genes may escape inactivation with aging, it is possible that autophagy flux is altered after ischemic stroke in a sex-dependent manner [86]. This may be more prominent in older animals, and future studies will be needed to determine if stroke-induced autophagy is altered with aging, and if this occurs differentially by sex.

## 5. Conclusions

**Figure 6:**
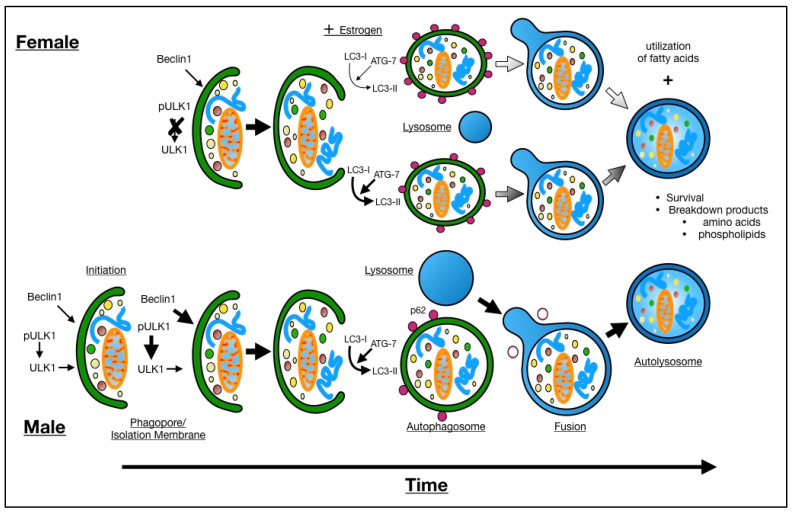
Schematic of sex differences in the autophagy pathway.

This study shows that induction of autophagy in the ischemic brain differs between the sexes both in vivo and in vitro (Figure 6). Furthermore, we show the sex specific differences of 3MA treatment and that estrogen may in part be responsible for these outcomes. These data may have important implications for the development of therapeutics. The FDA has recognized the importance of considering sex in development of therapeutic targets for over two decades [87,88]. These results reinforce this concept [72].

## Data Availability

The data sets generated during the current study are available from the corresponding author on reasonable request.

## Supplementary Materials

The following are available online at https://www.mdpi.com/article/10.3390/cells10071825/s1, Figure S1: Sex-specific cultures of primary neurons, Figure S2: LC3 levels in the brains of mice 72 h of MCAO, Figure S3: Co-localization of p62 and LC3 in neurons in the peri-infarct cortical region, Figure S4: Neuronal survival in male and female neurons 0 h and 120 h subjected to normoxia or OGD with or without E2.

## Abbreviations

CV	cresyl violet
DMSO	dimethyl sulfoxide
E2	estradiol
MCAO	middle cerebral artery occlusion
PARP-1	poly (ADP-ribose) polymerase
OVX	ovariectomy
3MA	3-methyladenine
