# Supplementary material for: Sex-Specific Differences in Autophagic Responses to Experimental Ischemic Stroke
Source: Cells. 2021 Jul 20;10(7):1825. doi: 10.3390/cells10071825 (PMC8304137; doi:10.3390/cells10071825)
Supplement: Supplementary file 1 [file cells-10-01825-s001.zip › cells-1254253-supplementary.pdf]

Supplemental Material

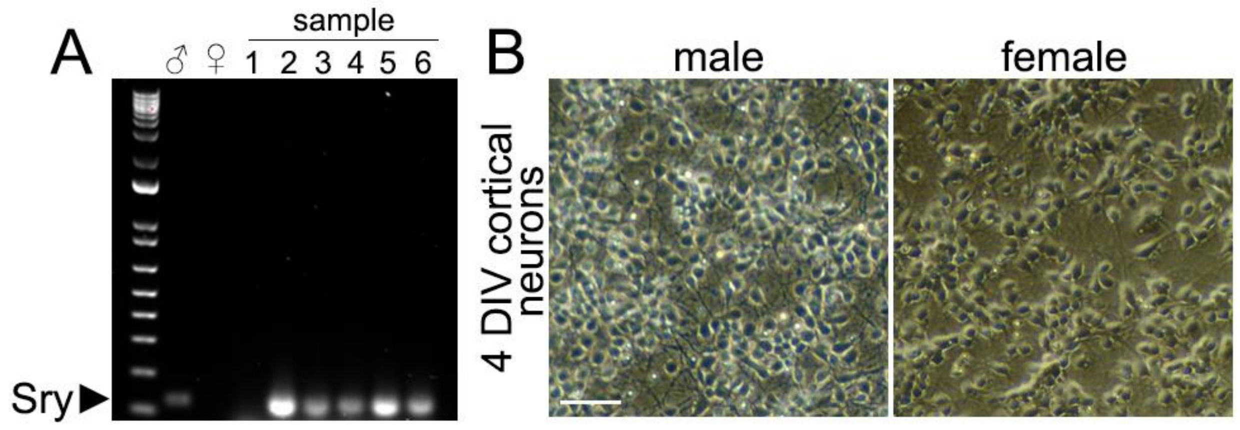

Supplemental Figure S1. Sex-specific cultures of primary neurons.

Supplemental Figure S1. Sex-specific cultures of primary neurons. **(A)** Sex determination by agarose gel electrophoresis using PCR primers for the Sry gene. This is a representative image of one of the gels for sex genotyping. The first lane at the left indicates the DNA ladder, the second and the third lanes correspond to positive and negative controls, respectively, and the subsequent lanes correspond to six samples. The figure shows that the sample 1 corresponds to a female embryo, while samples 2-6 are males. (**B**) Representative images of neuronal culture 4 days in vitro (DIV) after brain dissection from a male and a female E20 embryos. Scale bar, 50 µm.

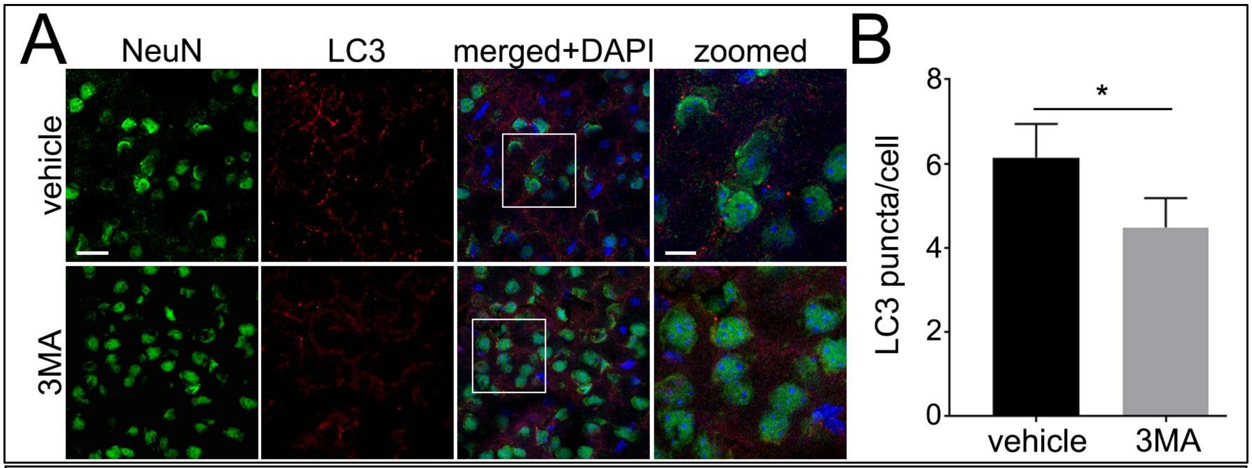

Supplemental Figure S2. LC3 levels in the brains of mice 72 h after MCAO.

Supplemental Figure S2. LC3 levels in the brains of mice 72 h after MCAO. (**A**) Representative images of vehicle and 3MA treated male stroke mice 72 hours after stroke onset. Brain sections were stained with antibodies against NeuN, and against LC3, and the nuclear Hoechst dye (DAPI). Scale bar, 25 µm; scale bar (zoomed), 10 µm. (**B**) Quantification of the number of LC3 puncta per NeuN-positive cell in the ipsilateral peri-infarct cortex. Unpaired t-test vehicle vs. 3MA (6.15±0.39 vs. 4.45±0.35, p=0.0203). 4 mice per group were analyzed. * Represents statistical significance ($p < 0.05$).

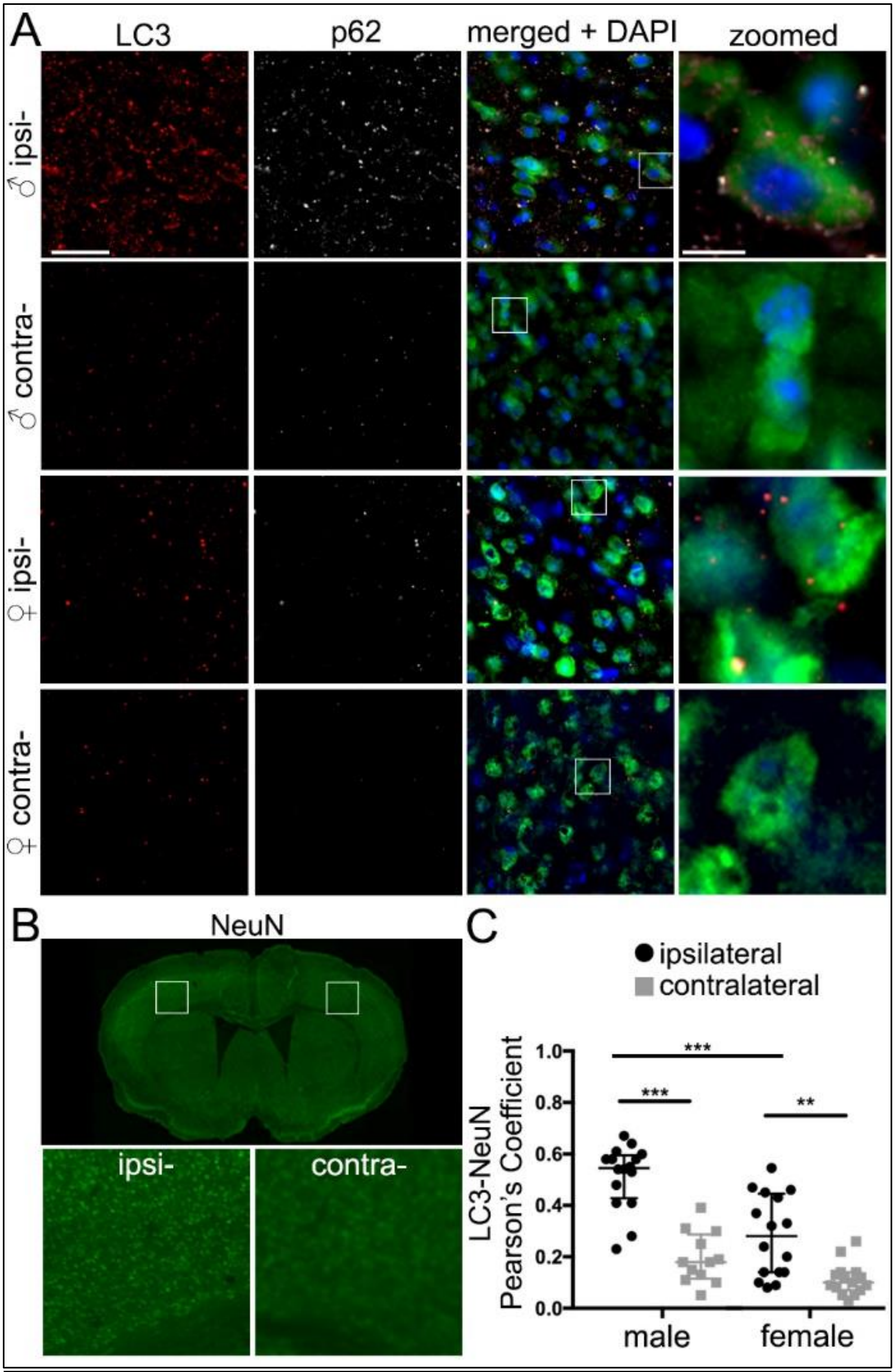

Supplemental Figure S3. Co-localization of p62 and LC3 in neurons in the peri-infarct cortical region.

Supplemental Figure S3. Co-localization of p62 and LC3 in neurons in the peri-infarct cortical region. (**A**) Representative 20X images of LC3 (red) and p62 (white) co-localize (pink) in neurons (green) in the ipsilateral and contralateral hemispheres 24 hours after stroke in male and females. Scale bar, 50 μm; scale bar (zoomed), 10 μm. (**B**) Representative brain slice depicting ROI of ipsilateral peri-infarct cortex and contralateral cortex. Quantification of LC3 within neurons. Pearson's correlation coefficient male ipsilateral vs. contralateral (0.51±.03 vs. 0.19±.02, p<0.0001), female ipsilateral vs. contralateral (0.23±.03 vs. 0.11±.01, p=0.001). （**C**）Male ipsilateral vs. female ipsilateral (p<0.0001). ** Represents statistical significance ($p < 0.001$), *** Represents statistical significance ($p < 0.0001$).

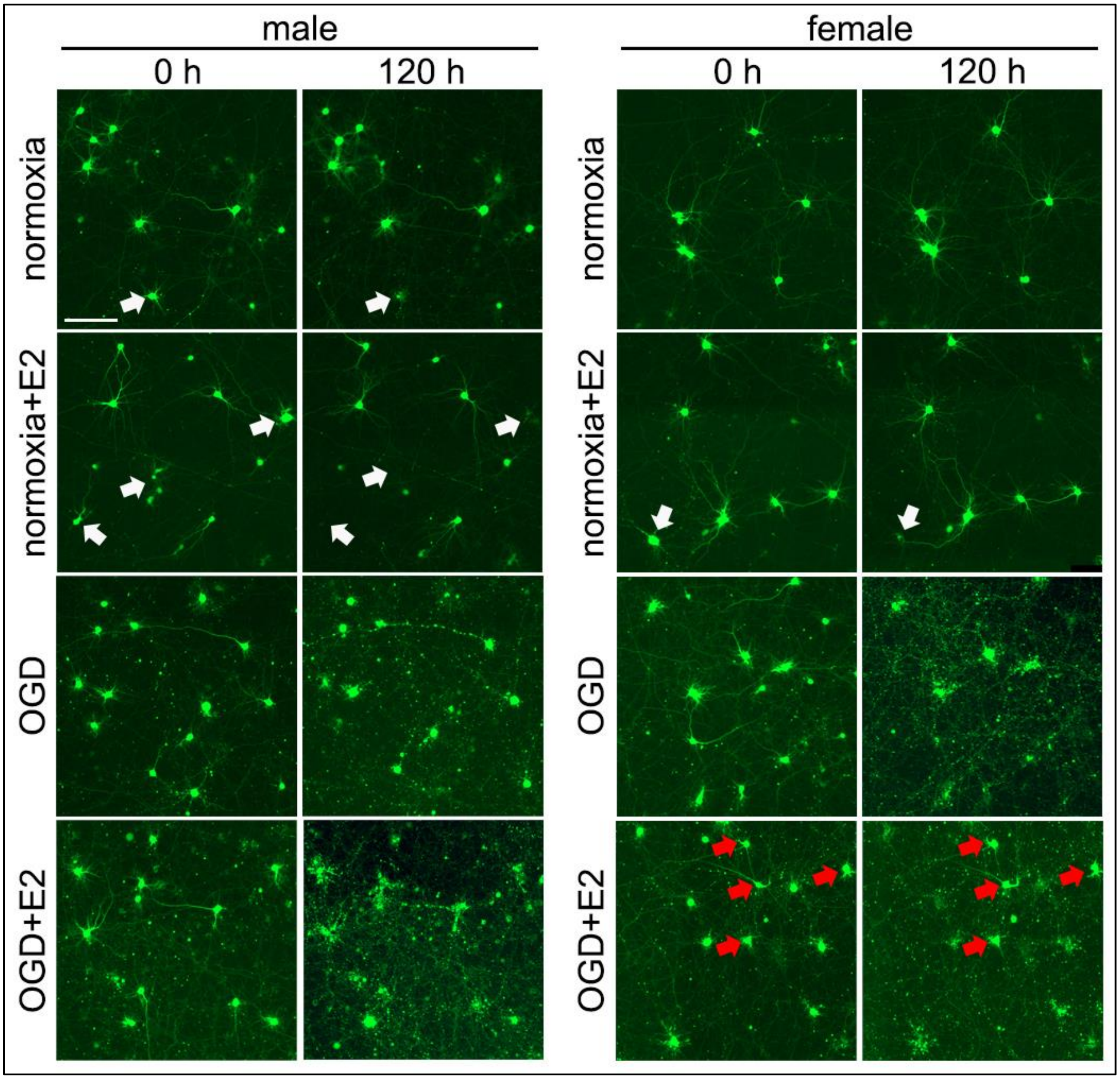

Supplemental Figure S4. Neuronal survival in male and female neurons 0 h and 120 h subjected to normoxia or OGD with or without E2.

Supplemental Figure S4. Neuronal survival in male and female neurons 0 h and 120 h subjected to normoxia or OGD with or without E2. Male and female GFP-transfected neurons were pre-treated with 17β-estradiol (E2) or a vehicle (ethanol) for 1 h, and then subjected to oxygen and glucose deprivation (OGD) or normoxia for 1 h. Supplemented neurobasal media was restored and cells were imaged immediately (0 h) and 5 days later (120 h). White arrows depict neurons subjected to normoxia±E2 conditions that died before 120 h. Note that less female neurons died during the experiment in normoxia conditions when incubated with E2. Red arrows depict neurons that remained alive 120

h after OGD+E2. Note that female neurons were more resistant to OGD when incubated with E2 compared with male neurons. Scale bar, 50 μm.
